# Viral Load and Cell Tropism During Early Latent Equid Herpesvirus 1 Infection Differ Over Time in Lymphoid and Neural Tissue Samples From Experimentally Infected Horses

**DOI:** 10.3389/fvets.2020.00621

**Published:** 2020-09-04

**Authors:** Kim S. Giessler, Susanna Samoilowa, Gisela Soboll Hussey, Matti Kiupel, Kaspar Matiasek, Dodd G. Sledge, Friederike Liesche, Jürgen Schlegel, Robert Fux, Lutz S. Goehring

**Affiliations:** ^1^Equine Hospital, Division of Medicine and Reproduction, Center for Clinical Veterinary Medicine, Ludwig-Maximilians University, Munich, Germany; ^2^Department of Pathobiology and Diagnostic Investigation, College of Veterinary Medicine, Michigan State University, East Lansing, MI, United States; ^3^Veterinary Diagnostic Laboratory, College of Veterinary Medicine, Michigan State University, Lansing, MI, United States; ^4^Section of Clinical and Comparative Neuropathology, Centre for Clinical Veterinary Medicine, Ludwig-Maximilians University München, Munich, Germany; ^5^Department of Neuropathology, School of Medicine, Institute of Pathology, Technical University Munich, Munich, Germany; ^6^Veterinary Science Department, Institute of Infectious Diseases and Zoonoses, Ludwig-Maximilians University, Munich, Germany

**Keywords:** EHV-1, horses, latency, Alphaherpesviruses, pathogenesis, trigeminal ganglia, lymphocytes

## Abstract

Upper respiratory tract infections with Equid Herpesvirus 1 (EHV-1) typically result in a peripheral blood mononuclear cell-associated viremia, which can lead to vasculopathy in the central nervous system. Primary EHV-1 infection also likely establishes latency in trigeminal ganglia (TG) via retrograde axonal transport and in respiratory tract-associated lymphatic tissue. However, latency establishment and reactivation are poorly understood. To characterize the pathogenesis of EHV-1 latency establishment and maintenance, two separate groups of yearling horses were experimentally infected intranasally with EHV-1, strain Ab4, and euthanized 30 days post infection (dpi), (*n* = 9) and 70 dpi (*n* = 6). During necropsy, TG, sympathetic trunk (ST), retropharyngeal and mesenteric lymph nodes (RLn, MesLn) and kidney samples were collected. Viral DNA was detected by quantitative PCR (qPCR) in TG, ST, RLn, and MesLn samples in horses 30 and 70 dpi. The number of positive TG, RLn and MesLn samples was reduced when comparing horses 30 and 70 dpi and the viral copy number in TG and RLn significantly declined from 30 to 70 dpi. EHV-1 late gene glycoprotein B reverse transcriptase PCR and IHC results for viral protein were consistently negative, thus lytic replication was excluded in the present study. Mild inflammation could be detected in all neural tissue samples and inflammatory infiltrates mainly consisted of CD3+ T-lymphocytes (T-cells), frequently localized in close proximity to neuronal cell bodies. To identify latently infected cell types, *in situ* hybridization (ISH, RNAScope®) detecting viral DNA was used on selected qPCR- positive neural tissue sections. In ganglia 30 dpi, EHV-1 ISH signal was located in the neurons of TG and ST, but also in non-neuronal support or interstitial cells surrounding the neuron. In contrast, distinct EHV-1 signal could only be observed in neurons of TG 70 dpi. Overall, detection of latent EHV-1 in abdominal tissue samples and non-neuronal cell localization suggests, that EHV-1 uses T-cells during viremia as alternative route toward latency locations in addition to retrograde neuronal transport. We therefore hypothesize that EHV-1 follows the same latency pathways as its close relative human pathogen Varicella Zoster Virus.

## Introduction

Equid Herpesvirus 1 (EHV-1) belongs to the family of Alphaherpesvirinae and is a clinically important herpesvirus in horses that causes respiratory disease, myeloencephalopathy and abortions worldwide. EHV-1 is typically spread by direct horse-to-horse contact through respiratory tract secretions and replicates in the upper respiratory tract epithelium following initial infection. Subsequently, EHV-1 infects mononuclear cells, enters the blood circulation and is thereby disseminated widely to secondary infection sites, such as the central nervous system (CNS) or the uterus (1–3). The main cell type infected during cell-associated viremia are monocytes and T-lymphocytes (T-cells), followed by B-lymphocytes (B-cells) (4–7).

During or shortly after acute lytic infection and replication, the virus starts to establish a life-long chronic persistent infection in the host. This latent infection is an evolutionary advantage found in all alphaherpesviruses, as it allows the virus to remain in the host undetected from the immune system while maintaining its capability for reactivation (8–10). A successful latent infection is described to be strongly dependent on a well-balanced host-virus interaction (11, 12), and neurons are thought to be excellent locations for latency establishment, as they represent a stable, long-living cell population. Furthermore, neurons are not known to express viral antigen, which is an important advantage for the virus allowing it to evade the immune system (3). During latency, viral DNA is present in the nucleus of infected cells in a non-integrated form, without lytic transcription and translation processes (13–15). In addition, there is growing evidence, that T-cells and satellite cells surrounding neurons are playing key roles in maintaining Alphaherpesvirus latency supposedly by interacting directly with viral transcription (12, 16).

EHV-1 has been shown to establish latency in the trigeminal ganglia (TG), like its relatives Herpes Simplex Virus 1 (HSV-1), Bovine Herpesvirus 1 (BoHV-1) and Varicella Zoster Virus (VZV) (8, 10, 12, 17) but also in respiratory associated lymphoid tissue (RALT) and circulating CD8+ T-cells (18–22). Furthermore, our research group has recently shown that 70 days post infection (dpi), chronic persistent EHV-1 infection is not limited to TG and RALT, but EHV-1 DNA can also be detected (via qPCR) in various other sensory- (spinal cord dorsal root ganglia), sympathetic and parasympathetic- ganglia as well as in abdominal lymphoid tissue (mesenteric lymph node, spleen) (23). We reported wide distribution of chronic persistent EHV-1 infection beyond viremia. Important remaining questions are how the virus gets to these sites of presumed latency, and which cells predominantly harbor the virus at these locations; e.g., which cells are preferred for latency establishment.

Most Alphaherpesviruses gain access to the sensory nerve endings in the vicinity of the primary infection sites and use retrograde axonal transport to reach the neuronal cell bodies. For VZV, which, like EHV-1, belongs to the subfamily of Varicellovirinae, it is thought that VZV is transported to the sensory ganglia during viremia and in VZV-infected T-cells, as well as by axonal transportation (24, 25). EHV-1 tropism for T-cells is also well described and it is proposed to be important for viral dissemination throughout the host and for bypassing viral clearance by the immune system (7, 22, 26). Interestingly, while EHV-1 gene transcription and protein expression is active during viral replication in epithelial cells, during viremia, the virus seems to be carried in a non-replicative state, until secondary locations, such as CNS or uterine endothelium, are reached and virus transfer occurs (7). However, it is unclear if EHV-1 is also transported to ganglia in the same manner and uses this route for successful latency establishment.

In the present study, we compared EHV-1 viral load and state of infection in neural and lymphatic tissue (based on previously identified locations at 70 dpi) (23) with a timepoint closer to acute infection and viremia (30 dpi). We hypothesized that EHV-1 chronic persistent infection is influenced by time passed after inoculation. Furthermore, we wanted to investigate latency establishment and maintenance on a cellular level by using *in situ* hybridization (ISH) to localize viral DNA within neural tissue.

## Materials and Methods

### Animals

Tissue samples used in this study were obtained from a total of fifteen yearling horses of both sexes (six females and nine males). All horses were clinically healthy and screened for EHV-1 and EHV-4 serum neutralizing antibodies using serum neutralization (SN) tests prior to infection. Only horses with a titer <4 for EHV-1 and <40 for EHV-4 were selected for the study. Animals were fed twice a day and had *ad libitum* access to water. Horses were housed together in a naturally ventilated barn throughout the experiment. The maintenance and experimental protocols were reviewed and approved by Michigan State University Institutional Animal Care and Use Committee.

### Experimental Design

Two separate experimental groups of horses were established (group 1: *n* = 9, group 2: *n* = 6) and infected by intranasal instillation of 5 × 10^7^ plaque forming units (PFU) of EHV-1 in 10 ml of saline of the neuropathogenic EHV-1 strain Ab4 (D752 variant). This virus was previously isolated from a quadriplegic mare (27). Horses in group 1 were euthanized 30 dpi, while horses in group 2 were euthanized 70 dpi.

### Clinical Data, Nasal Virus Shedding, and Viremia

Collection of clinical data, nasal viral shedding and viremia are described in detail elsewhere (28). Briefly, clinical examinations were performed and nasal swabs for viral DNA isolation were collected prior to infection (at day −5 and −3) and daily from day 1 to 14, and every other day from day 14 to 21 dpi. Blood samples were taken prior to infection (at day −5) and daily from 1 to 10 dpi for detection of cell-associated viremia. Nasal viral shedding and viremia were analyzed by real-time PCR (qPCR) using specific probes and primers for EHV-1 glycoprotein B (1).

### Necropsy and Tissue Collection

All horses were euthanized by sedation with detomidine (0.012 mg/kg, i.v.) followed by administration of pentobarbital (380 mg/kg, i.v.) and immediately (within 10–15 min) transported to the Veterinary Diagnostic Laboratory at MSU for necropsy and sample collection. Abdominal sympathetic trunk ganglia (ST), TG, retropharyngeal lymph nodes (RLn) and mesenteric lymph nodes (MesLn) were collected from all animals. For two horses (one in each infection group), RLn samples were not available for the study. Furthermore, kidneys were collected from all animals as negative tissue controls, hypothesizing, that this sample type represents no target for EHV-1. Time from euthanasia to completed tissue collection was <70 min. All tissues were fixed in 5% paraformaldehyde for 24 h, followed by routine tissue processing and paraffin embedding.

### DNA/RNA Extraction

Viral DNA and total RNA from paraformaldehyde-fixed-paraffin-embedded (PFPE) tissue was extracted using AllPrep DNA/RNA FFPE Kit (Qiagen, Hilden, Germany). Briefly, five serial sections of 4 μm (20 μm in total) were used and processed according to the manufacturer's protocol. Extraction from DNA-free and RNA-free water was included as quality control during extraction. For neural tissue, Hematoxylin and Eosin (H&E) slides were screened for the presence of ganglia cell bodies before DNA/RNA isolation. All RNA extraction samples were tested with qPCR for absence of genomic DNA using primers and probe for equine glyceraldehyde-3-phosphate dehydrogenase (eqGAPDH) as previously published (29). Genomic DNA positive RNA samples were treated with the RQ1 Rnase-free Dnase Kit (Promega, Madison, WI, USA) according to the manufacturer's instructions and re-tested afterwards, as described above. Only samples negative for genomic DNA were converted to complementary DNA (cDNA) using the Quantinova Reverse Transcription Kit (Qiagen, Hilden, Germany) with random hexamer primers according to the manufacturer's protocol.

### Real-Time PCR

For detection of EHV-1 genomic DNA and late gene mRNA, a qPCR assay targeting a 106 bp long region of the glycoprotein B gene (open reading frame 33) was performed as previously published (1). Forward and reverse primers were used in a final concentration of 450 nM and the probe in a final concentration of 100 nM. Sequences for primers and probes used in this study are listed in Table 1.

**Table 1:** Primers and probes for qPCR used in this study.

**eGAPDH**	
egapdh (F)	5^′^- GCCATCACTGCCACCCAG-3^′^
egapdh (R)	5^′^- TGGCAGCACCAGTAGAAGCA-3^′^
egapdh (probe)	5^′^[FAM]- AGGGGCTGCCCAGAACATCATCC - [TAMRA]3^′^
**B2M**	
B2M (F)	5^′^-ATGGAAAGCCAAATTTCCTG-3^′^
B2M (R)	5^′^-ACCGGTCGACTTTCATCTTC-3^′^
B2M (probe)	5^′^[HEX]-TGGGTTCCATCCGCCTGAGA –[BHQ1]3^′^
**gB**	
gB (F)	5^′^-CATACGTCCCTGTCCGACAGAT-3^′^
gB (R)	5^′^-GGTACTCGGCCTTTGACGAA-3^′^
gB (probe)	5^′^[FAM]- GGTACTCGGCCTTTGACGAA -[BHQ1]3^′^

All qPCR reactions were performed in a total reaction volume of 20 μl using 10 μl 1 × SensiFAST™ Probe Lo-ROX Kit (Bioline, Luckenwalde, Germany) and 5 μl of the template. All samples were analyzed in duplicates and amplified with the following thermal profile: initial 95°C step for 2 min, followed by 40 cycles of 95°C for 10 s and 60°C for 60 s (and hold 60°C for 60 s).

For absolute quantification, DNA and mRNA results were compared to a standard curve generated with cloned EHV-1 oligonucleotides that were kindly provided by W. Azab and N. Osterrieder. Viral copy numbers were then normalized to a standard curve generated with oligonucleotides specific to the housekeeping gene equine B2M, as previously described (30). Viral DNA and mRNA concentrations were expressed as copies per 10^6^ cells, considering that each diploid eukaryotic cell contains two copies of B2M gene (30). Positive EHV-1 DNA (extracted from lung of an EHV-1 aborted fetus) and horse DNA (extracted from equine liver) were included as positive controls for EHV-1 gB and B2M qPCR assay. DNA-free and RNA-free water was used as negative control.

### Hematoxylin and Eosin Staining & Histology Grading

For Histological evaluation, 4 μm thick sections of the PFPE blocks were routinely stained with Hematoxylin and Eosin (H&E). Ganglion cells, satellite cells and nerve fibers were evaluated for the presence of histopathological changes and inflammatory infiltrations were assessed and scored as mild, moderate or severe, if present.

### Immunohistochemistry

Immunohistochemistry (IHC) was conducted on samples positive for EHV-1 genomic DNA using EHV-1/EHV-4 polyclonal caprine antiserum (VMRD, Pullman, USA) cross-reactive with EHV-1 and EHV-4 antigen (Ag). Serial section of PFPE blocks were deparaffinized and antigen retrieval was performed by incubation in citrate buffer (0.1 M, pH6.0) and heating in a microwave oven (700 W) for 20 min. Endogenous peroxidase was quenched through incubation with H_2_O_2_ (1%, 15 min) and subsequently nonspecific binding of proteins was blocked with blocking buffer containing rabbit serum (1:10, 30 min), followed by incubation with the primary antibody (Ab) against EHV-1/EHV-4 Ag (VMRD, Pullman, USA, polyclonal, goat, 1:1,600) for 1 h at room temperature. Subsequently, samples were incubated with rabbit anti-goat biotinylated Ab (Vector laboratories LTD, Burlingame, USA), then incubated with avidin-biotin-complex (1:100, 30 min) and visualized by 3,3'- diaminobenzidine (DAB) (Vector laboratories LTD, Burlingame, USA). Counterstaining was performed with Mayers Hemalum. Lung tissue from an EHV-1 aborted fetus was included as positive control. As a negative control, the primary Ab was omitted and replaced by the corresponding antibody dilutent (blocking buffer containing rabbit serum). Non-neuronal cells were characterized on selected neural samples using antibodies directed against CD3 (DAKO, Hamburg, Germany, polyclonal, rabbit, 1:500), CD20 (Thermo Scientific Labvision, Fremont CA, USA, polyclonal, rabbit, 1:1,000) and S-100 (DAKO, Hamburg, Germany, polyclonal, rabbit, 1:6,000) using routine methods. Briefly, pre-treatment was performed as described for EHV-1/EHV-4 with blocking buffer containing goat serum, followed by incubation with the correspondent primary Ab overnight at 4°C. Incubation with goat anti-rabbit biotinylated Ab, detection and counterstaining was performed as described for EHV-1/EHV-4 Ab.

### *In situ* Hybridization

*In situ* hybridization detecting viral DNA was performed by RNAScope® technology (Advanced Cell Diagnostics, Inc., USA) as previously described (31–33) using the RNAScope® 2.5 Detection Kit (Red) and the EHV-1 probe (V-EHV-1-ORF33, Cat.no.552651) targeting the region between nucleotides 61485-62416 of AY665713.1. Briefly, 4 μm serial sections were cut from PFPE blocks of selected EHV-1 qPCR positive TG (group 1, *n* = 3, group 2, *n* = 4) and ST (group 1, *n* = 4; group 2, *n* = 3). Subsequently, deparaffinization, pretreatment and hybridization were performed according to the manufacturer's protocol using the provided pretreatment solutions and wash buffer. All incubation steps were performed in a humidity control tray and a HybEZTM oven (Advanced Cell Diagnostics, Inc., USA). Following hybridization the signal was detected using Fast Red as chromogen provided by the manufacturer (RedB:RedA, 1:60 ratio). Counterstaining was performed using 50% Gill's Hematoxylin 1 (American MasterTech, Lodi, CA), followed by bluing with tap water and 0.02% ammonium hydroxide water. Slides were air dried and mounted with Xylene and EcoMount (EcoMount, Biocare Medical, Concord, CA).

EHV-1 qPCR positive (lung of an EHV-1 aborted fetus) and qPCR negative (TG) tissues were used as controls and an EHV-1-scrambled probe was designed targeting the same region as the target probe but with minor sequence alteration. For technical assay control, the housekeeping gene Peptidylprolyl isomerase B (PPIB) was used as positive control and the bacterial gene dihydrodipicolinate reductase (dapB) was included as negative control according to the manufacturer's protocol.

### Statistical Analysis

Statistical analysis was performed using GraphPad Prism version 6.01 for Windows (GraphPad Software, La Jolla California USA, www.graphpad.com). Differences in the number of EHV-1 qPCR positive samples between the two infection groups were evaluated using Fisher's Exact Test. Mann-Whitney U test was used to compare significant differences in viral load between the two infection groups. *P*-values < 0.05 were considered statistically significant.

## Results

### Clinical Data, Viral Nasal Shedding, and Viremia

Animals in both infection groups showed respiratory symptoms and a classical bi-phasic fever post infection. Viral DNA amounts in nasal shedding and cell-associated viremia were detected in both groups for multiple days post infection. Overall, no significant differences were observed when comparing the two groups (data not shown) (28).

### Detection of EHV-1 gB DNA and Characterization of Viral Activity

EHV-1 DNA was detected at both timepoints post infection in TG, ST, RLn, and MesLn samples. Viral distribution and -load for both groups are shown in Table 2. Kidney samples were consistently negative for EHV-1 DNA in both groups (data not shown). Lytic replication of EHV-1 was excluded for each EHV-1 DNA positive sample using reverse transcription PCR (RT-PCR) for gB mRNA and IHC for viral antigen. Specific gB mRNA amplification signal or viral antigen could not be detected in EHV-1 DNA positive tissue samples. Therefore, we concluded that there was no lytic EHV-1 infection in any of the examined tissue samples. Two out of 15 retropharyngeal lymph nodes were not available for this study and for horses 70 dpi, 3/6 sympathetic trunk ganglia samples could not be included in further analysis due to the absence of neural cell bodies in the collected tissue sample.

**Table 2:** Distribution of EHV-1 genomic DNA 30 and 70 days post infection (dpi) (EHV-1 gDNA copies/10^6^ cells).

**dpi**	**Horse ID**	**TG**	**ST**	**RLn**	**MesLn**
30	1,598	–	–	1.43 x 10^4^	3.81 x 10^2^
	1,619	1.42 × 10^4^	–	2.19 × 10^3^	6.35 × 10^1^
	1,620	3.13 × 10^3^	–	7.72 × 10^1^	7.89 × 10^2^
	1,651	5.41 × 10^3^	1.98 × 10^5^	n.d.	6.64 × 10^3^
	1,621	2.58 × 10^4^	–	1.40 × 10^3^	6.85 × 10^2^
	1,636	1.10 × 10^4^	–	7.30 × 10^2^	1.43 × 10^2^
	1,638	2.03 × 10^3^	1.80 × 10^4^	7.53 × 10^2^	2.81 × 10^2^
	1,628	3.05 × 10^3^	3.27 × 10^4^	3.63 × 10^3^	6.45 × 10^1^
	1,629	4.03 × 10^3^	5.07 × 10^3^	1.97 × 10^2^	–
70	905	4.19 × 10^1^	3.82 × 10^4^	–	–
	909	–	n.d.	n.d.	–
	913	–	–	–	1.72 × 10^3^
	900	9.93 × 10^1^	n.d.	–	–
	914	2.11 × 10^1^	1.57 × 10^4^	2.62 × 10^2^	1.47 × 10^3^
	908	3.51 × 10^1^	2.55 × 10^3^	–	7.48 × 10^2^

*–, negative; n.d., not determined; TG, trigeminal ganglia; ST, sympathetic trunk ganglia; RLn, retrophrayngeal lymph node; MesLn, mesenteric lymph node*.

### Comparison of Viral Distribution and Copy Number at 30 and 70 dpi

**Figure 1:**
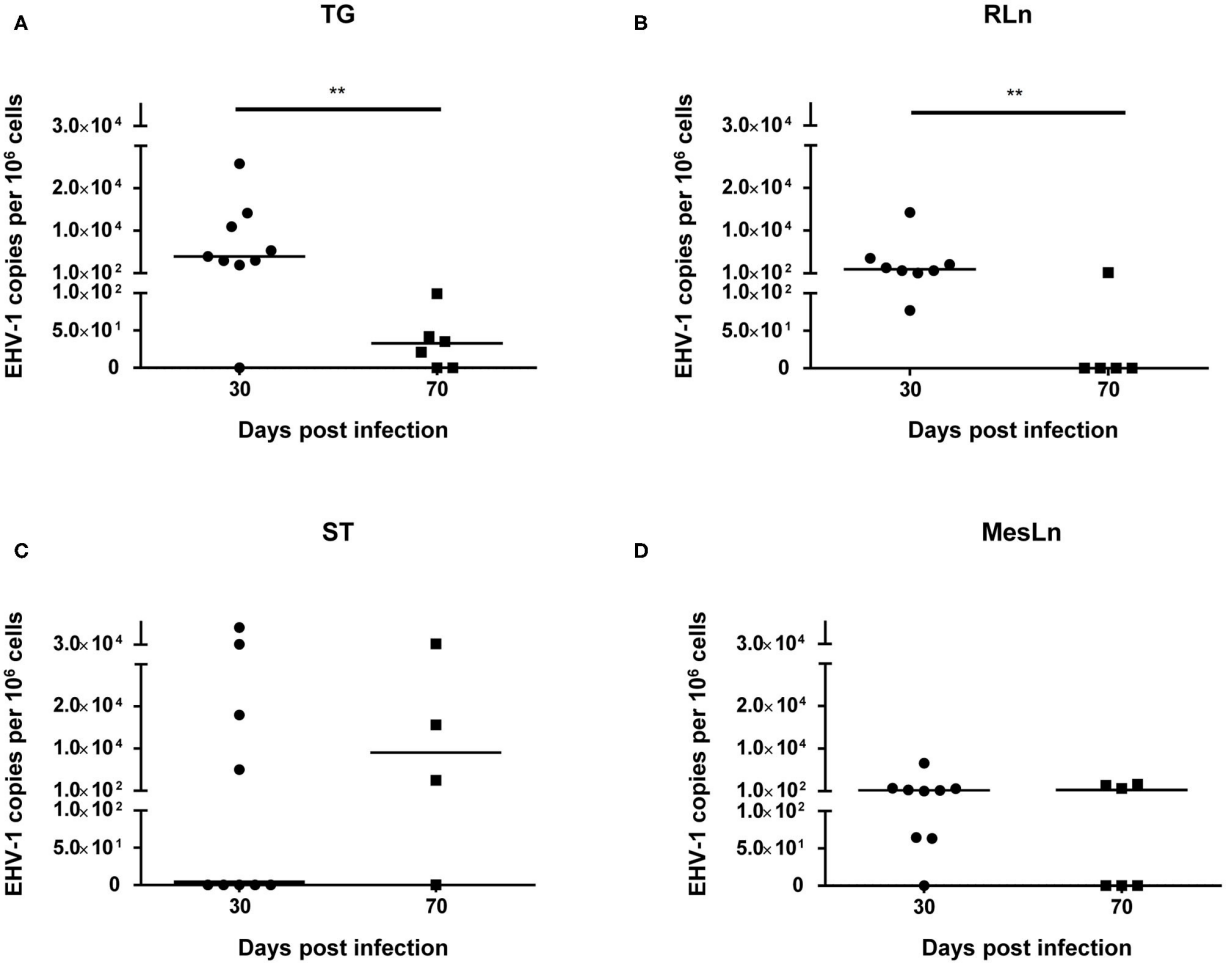
Significant differences (**P < 0.01) in viral copy numbers in **(A)** trigeminal ganglia and **(B)** retropharyngeal lymph nodes at 30 dpi compared to 70 dpi. Viral copy numbers in **(C)** sympathetic trunk and **(D)** mesenteric lymph nodes at 30 dpi compared to 70 dpi. Line indicates the median.

When comparing samples from horses 30 dpi with samples from horses 70 dpi (Table 2), a reduction of the percentage of positive tissue samples could be detected for TG, RLn, and MesLn samples, but this difference was only statistically significant for RLn samples. All of the available RLn samples at day 30 pi were positive (8/8), while only 1/5 was positive at day 70 pi. There were 8/9 positive TG and MesLn samples 30 dpi compared to 4/6 and 3/6 positive samples by 70 dpi, but the difference in percentage of positive samples for this group was not statistically significant. For ST, 4/9 in the 30 dpi group were positive for EHV-1 DNA and 3/4 samples were positive at day 70 pi, but this difference was not statistically significant. The viral copy number in both TG and RLn samples was significantly higher at 30 dpi when compared to samples collected at 70 dpi (Figures 1A,B). No statistically significant difference in viral copy number could be observed in ST or MesLn samples (Figures 1C,D).

### Histopathological Evaluation and Cellular Characterization

**Figure 2:**
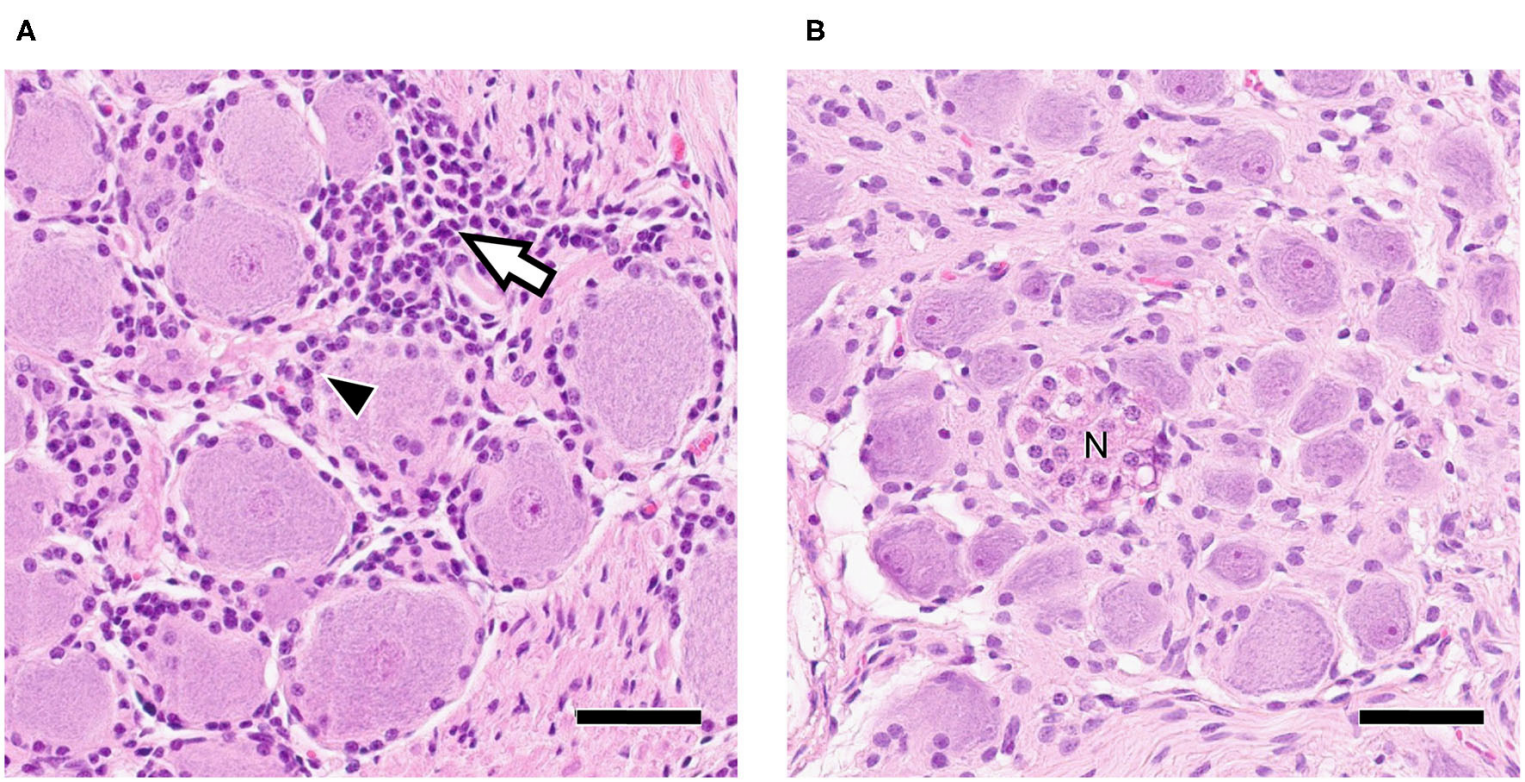
**(A)** Trigeminal Ganglion 30 dpi: Moderate infiltration of inflammatory cells (white arrow) and satellitosis (arrowhead); **(B)** Sympathetic trunk ganglion 30 dpi: Nageotte's body (N) as sign of neuronal degeneration; Bars = 50 μm.

**Figure 3:**
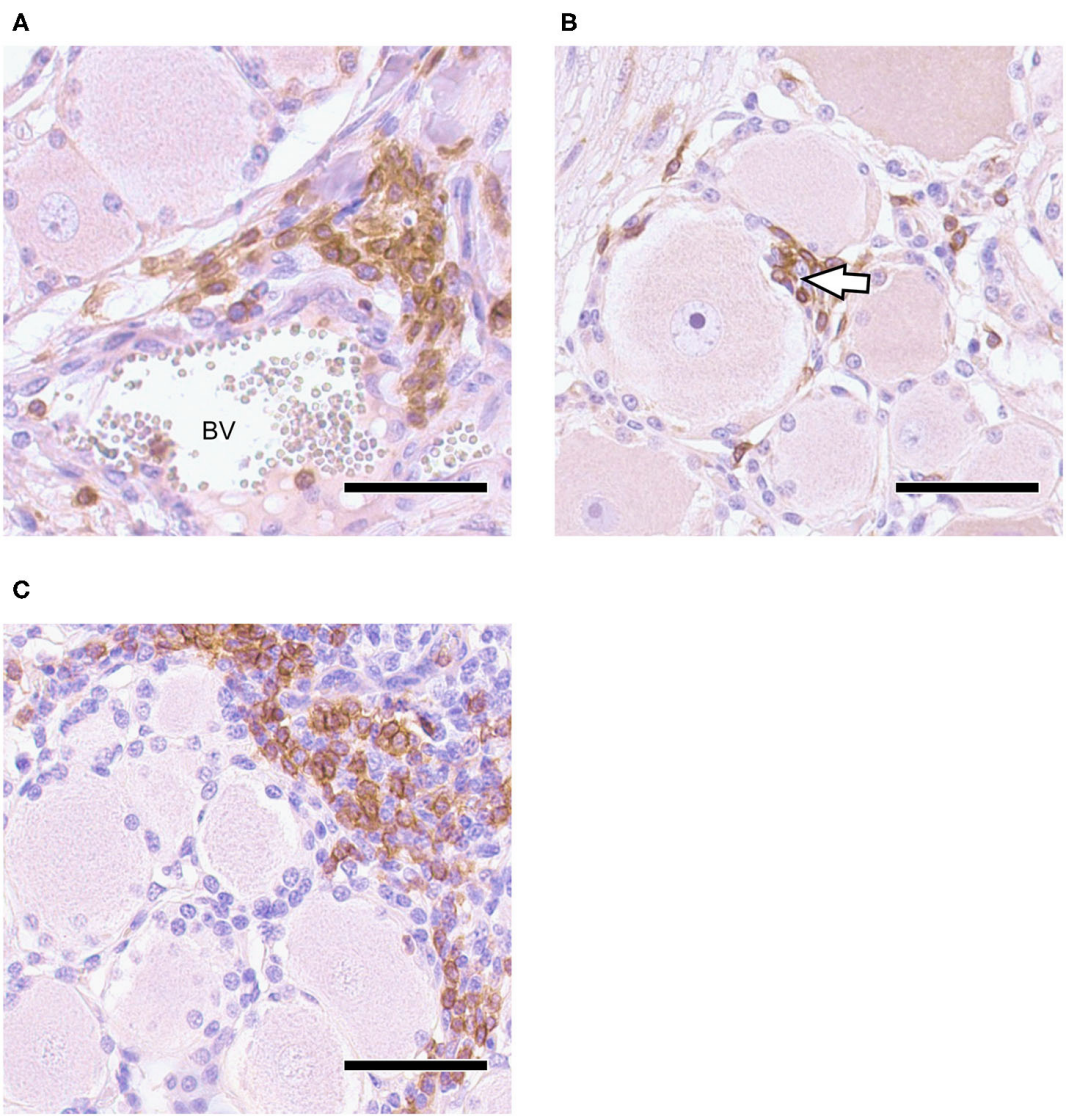
Trigeminal Ganglion 30 dpi, IHC for CD3 and CD20, DAB with Mayer's hemalum counterstaining: **(A)** CD3+ T-cell infiltration in vicinity of blood vessel (BV); **(B)** CD3+ T-cells penetrate neuron-satellite sheet (arrowhead); **(C)** localized infiltrates of CD20+ B-cells associated with non-labeling T-cells; bars = 50 μm.

Neural tissue sections were analyzed for histopathological changes at 30 and 70 dpi. All TG and 7/12 ST had mild inflammatory infiltrations and mild satellitosis (Figure 2A) at both 30 and 70 dpi. In addition, Nageotte's bodies, which are a sign for neuronal degeneration (34) were detected in TG and in ST occasionally in both groups but no differences in numbers were observed when comparing time points (Figure 2B). No differences in severity of inflammation could be observed when comparing the two groups. Further characterization of ganglia at 30 dpi by IHC revealed that inflammatory infiltrates consisted of mainly CD3+ T-cells (Figures 3A,B). Few CD20+ B-cells were detected (Figure 3C).

### Detection of EHV-1 gB DNA by *in situ* Hybridization

**Figure 4:**
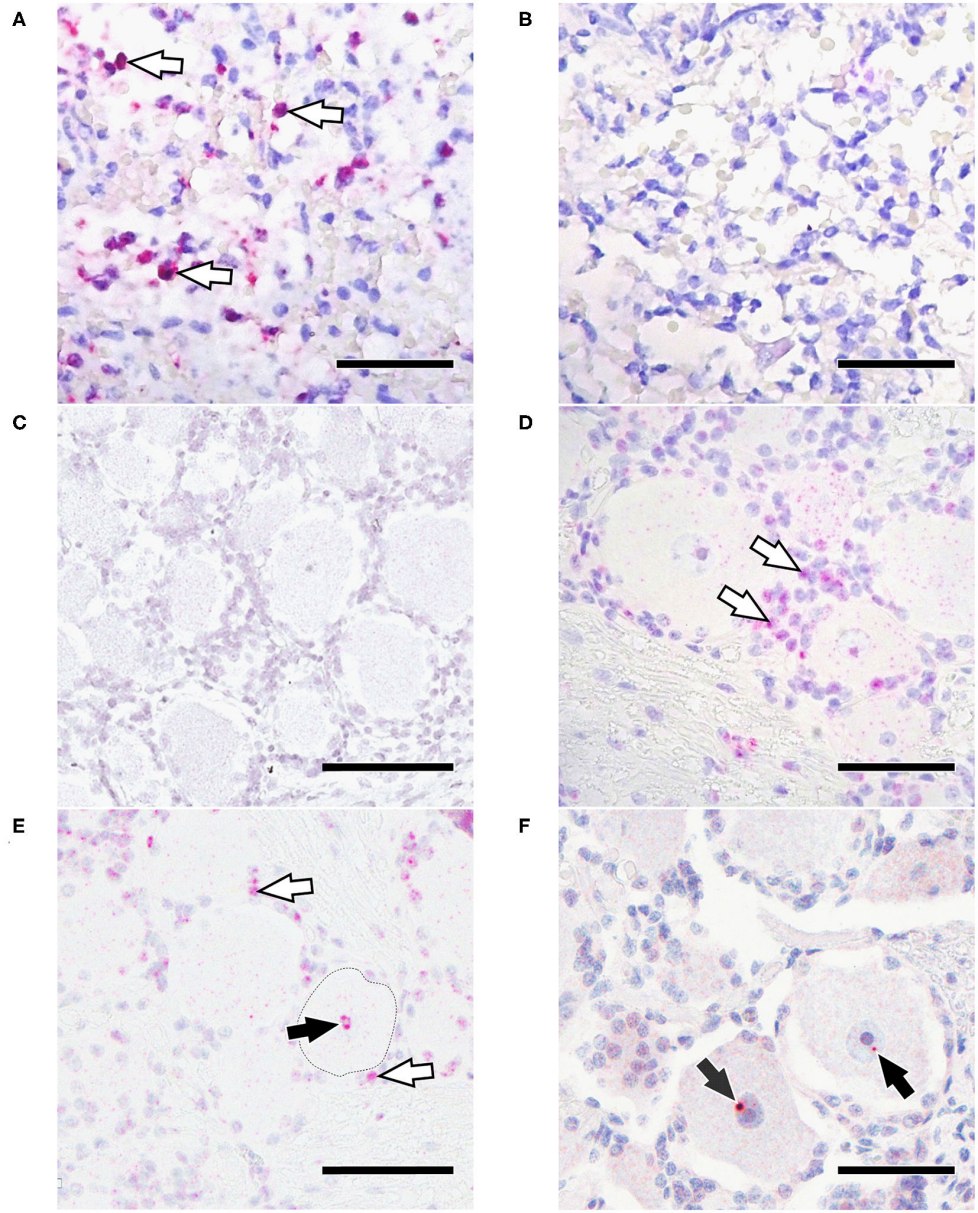
*In situ* hybridization for EHV-1 gB. **(A)** Positive control with positive labeling (arrowheads); **(B)** Positive control tissue with no signal using an EHV-1 scrambled probe; **(C)** TG from a EHV-1 qPCR negative horse lacks positive ISH signal; **(D)** EHV-1 qPCR positive TG 30 dpi with strong signal in non-neuronal cells (arrowheads); **(E)** EHV-1 qPCR positive TG 30 dpi with strong in non-neuronal cells (white arrowheads) and nucleus of the neuron (black arrowhead); **(F)** EHV-1 positive TG 70 dpi with positive signal in the nucleus of the neuron (arrowhead). Bars = 50 μm.

The EHV-1 gB probe detected large number of positive cells in the known EHV-1 positive control (Figure 4A), while the negative control probe (a.k.a. EHV-1 scrambled probe), showed no signal (Figure 4B). In addition, no signal was detected with the EHV-1 gB probe in a trigeminal ganglion from an EHV-1 negative horse (as determined by qPCR), which was euthanized for unrelated reasons (Figure 4C).

A positive signal could be detected in 6/7 TG samples and 4/7 ST samples that had tested positive for EHV-1 by qPCR. Results are shown in Table 3 and Figures 4D–F. At 30 dpi, all tested TG (3/3) had a positive ISH signal in ganglion cells as well as strong concurrent labeling in non-neuronal cells surrounding ganglia (Figures 4D,E). Within neurons, the ISH signal was located in the nucleus and the cytoplasm. For ST at 30 dpi, 3/4 were positive by ISH with signal being detected in the cytoplasm of neuronal cell bodies and in 2/4 samples also in non-neuronal cells. At 70 dpi, an EHV-1 hybridization signal was detected in the nuclei of ganglia cell bodies from 3/4 TG, but not in non-neuronal cells (Figure 4F). For ST, only 1/3 samples from horses euthanized at 70 dpi had a positive signal in the cytoplasm of the neurons but was also positive in non-neuronal cells.

**Table 3:** Cellular visualization of EHV-1 DNA in trigeminal ganglia and sympathetic trunk ganglia using *in situ* hybridization.

	**30 dpi**				**70 dpi**			
			**Viral localization (ISH)**				**Viral localization (ISH)**	
**Tissue**	**Horse ID**	**Viral load gDNA gB**	**Neuron**	**Non-neuronal cells**	**Horse ID**	**Viral load gDNA gB**	**Neuron**	**Non-neuronal cells**
TG	1,651	5.41 × 10^3^	+	+	905	4.19 × 10^1^	+	–
	1,621	2.58 × 10^4^	+	+	900	9.93 × 10^1^	+	–
	1,628	3.05 × 10^3^	+	+	914	2.11 × 10^1^	+	–
					908	3.51 × 10^1^	–	–
ST	1,651	1.98 × 10^5^	+	+	905	3.82 × 10^4^	+	+
	1,638	1.80 × 10^4^	–	–	914	1.57 × 10^4^	–	–
	1,628	3.27 × 10^4^	+	–	908	2.55 × 10^3^	–	–
	1,629	5.07 × 10^3^	+	+				

*Viral load expressed in EHV-1 gDNA copies/10^6^cells based on qPCR*.

*dpi, days post infection; ISH, in situ hybridization; TG, trigeminal ganglia; ST, sympathetic trunk ganglia; +, positive ISH signal; –, no ISH signal*.

## Discussion

This study compares neuropathogenic EHV-1 strain Ab4 distribution and persistence in selected lymphoid and neural tissues collected at different time points (30 and 70 days) following experimental infection. We detected EHV-1 in non-neuronal support or interstitial cells of trigeminal and sympathetic trunk ganglia. These findings provide new insight in EHV-1 latency establishment as we propose that in addition to retrograde neuronal transport, EHV-1 uses mononuclear cells during viremia as alternative route toward latency locations.

The current understanding is that EHV-1 latency is established in the trigeminal ganglia and respiratory associated lymphoid tissue during primary respiratory tract infection and/or during cell-associated viremia (8, 9, 21). Both of our sample collection timepoints were chosen after active viral replication and viremia had ceased in all horses. Typically, this occurs around 14 days after experimental infection (1, 35–38), which was also the case in all horses sampled for this study. Furthermore, at both collection time points, neither gB (late gene) mRNA, nor viral protein could be detected, favoring the idea that EHV-1 was present in a quiescent state in both EHV-1 DNA positive tissue sets, as defined previously (15). Thirty days post infection, EHV-1 (gB) DNA was detected at high copy number in TG, ST, RLn and in MesLn samples. While TG and RLn are considered the most common latency locations described in previous studies, we also show latent EHV-1 to be present in abdominal neuronal and lymphoid tissues. This also confirms our previous results, where we showed viral distribution in various neuronal and lymphoid tissues, and not just in the vicinity of primary infection at day 70 post infection (23). These findings raise the question of how the virus is transported to abdominal locations, as a thorough understanding of EHV-1 pathogenesis is crucial to further define the balance between latency and reactivation.

The pathogenesis of EHV-1 latency establishment is currently best studied for the trigeminal ganglia but overall poorly understood. EHV-1 is thought to follow the strategy of other Alphaherpesviruses and to reach neuronal cell bodies via retrograde axonal transport (3, 8, 9, 39), following primary infection of the upper respiratory tract. Alphaherpesviruses are known to enter sensory nerve endings and are transported actively by using cellular molecular motor proteins to the ganglion cell body (40). Here, the virus translocates to the nucleus of the neuron and becomes latent after a short initial lytic replication cycle (12). While this pathway seems to be likely for EHV-1 latency establishment in neuronal tissue in the vicinity of the primary infection sites, retrograde axonal transport to abdominal sympathetic trunk ganglia would be challenging as the virus would have to travel very long distances and potentially overcome multiple synapses. VZV, a close relative to EHV-1, establishes latency in TG and dorsal root ganglia (DRG) but also infects various sensory and autonomic ganglia (41–46). Recently, there is growing evidence that VZV most likely uses infected lymphocytes as alternative route to reach ganglion neurons during cell-associated viremia (24, 25, 47).

In order to get a better understanding of the pathways and cellular interplays of EHV-1 latency establishment, we used *in situ* hybridization to determine the cellular localization of latent EHV-1 DNA in TG and ST samples. While in samples collected 30 dpi a positive EHV-1 ISH signal was noticed in the neurons of TG and ST, a strong signal was also detected in the (non-neuronal) support and interstitial cells. These findings suggest that there exists an alternative pathway for EHV-1 latency establishment, which might be similar to VZV. Numerous studies described neurons as main infected cell types in ganglia during latency for HSV-1, BoHV-1 and VZV (48–51). However, only few studies tried to determine latent EHV-1 localization in ganglia. Baxi et al. claimed latent EHV-1 in trigeminal neurons by using RNA *in situ* hybridization to detect potential latency associated transcripts (9). Neurons represent a stable cell population where the virus is able to hide lifelong in the host (52, 53) without being detected by the immune system. The viral DNA translocates to the nucleus of the infected neuron and persists in a circular episome, not integrated into the host DNA. In addition to the nuclear localization of viral DNA, we could also detect ISH signal frequently in the cytoplasm of TG and ST neurons. This cytoplasmatic signal could not be detected in samples with the EHV-1 scrambled (negative) probe or with the technical assay control probe provided by the manufacturer. Therefore, unspecific probe binding seems unlikely. Similarly, cytoplasmatic signal of VZV DNA could be found in neurons, where the virus reactivates, and lytic infection occurs (54). IHC for lytic protein and for late gene (gB) mRNA could not be detected in the present study, and thus we exclude an overall active infection. However, latent infection is not exclusively silent and occasional transcription of lytic genes is described for HSV-1 and VZV latent infection at low levels (55). Therefore, we might have detected low levels of viral DNA or gB mRNA in the cytoplasm in the present study. This phenomenon needs further investigation, as we did not detect viral gB mRNA via RTqPCR and assume an overall higher sensitivity for the RTqPCR assay. However, RNAScope® assay has been described to be highly sensitive due to the unique selective amplification scheme in combination with a serial target probe design (31, 56).

To the best of our knowledge, EHV-1 has not been detected in non-neuronal (support or interstitial) cells within ganglia previously. Non-neuronal cells are mainly composed of glial cells of the peripheral nervous system (satellite glial cells) and mononuclear cells. Each neuronal cell body is enveloped by several satellite cells forming a functional unit. This is important for the regulation of extracellular chemical conditions of the neuron and providing physical support (34, 57). Furthermore, satellite cells act as antigen presenting cells during infections, attract T-cells and can interact with the neuron by signaling processes (57, 58). In acute human herpesvirus infections, the barrier formation of satellite cells around the neurons seems to protect the neuron from virus spread (57). However, it has been shown that this barrier is a flexible wall, where macrophages still can penetrate (59) and infiltrating T-cells tend to come in close contact to the neuron-satellite sheath (16). Furthermore, satellite cells are infected during productive VZV infection and seem to transfer virus to neurons by cell-to-cell fusions *in vivo* mouse models (60). To further differentiate lymphocytes from satellite cells in the present study, we used CD3+, CD20+ and S-100 IHC staining. We could show that besides local CD3+ and CD20+ lymphocytic inflammatory infiltrates, CD3+ T-cells also associate with satellite cells and tend to squeeze into the satellite sheath around neurons. This phenomenon was not detected for CD20+ B-cells. These results suggest that the EHV-1 ISH signal from non-neuronal cells surrounding neurons in the current study, might mainly attributed to T-cells and satellite cells.

T-cell tropism is well described for EHV-1 and suggested to be a key strategy for immune evasion and dissemination in the host (4, 7, 19, 21, 61, 62). During primary viral infection, EHV-1 seems to initially infect monocytes (4, 5, 63), while T-cells are the preferred cell type during viremia (6, 7, 61) to transport the virus to secondary target organs like the CNS or uterus. Among the circulating EHV-1 strains, the neurovirulent strain Ab4 has been shown to infect immune cells efficiently and rapidly following replication in the respiratory epithelium and subsequently result in a longer viremia with higher viral loads compared to non-neuropathogenic strain counterparts (5, 36, 64). It has been suggested that viral replication is not productive or restricted in carrier cells (65) or viral capsids are accumulated in the nucleus of the carrier cell, but viral egress is hampered until the endothelium of a secondary target organ is reached (7). This has also been described for VZV (11) and it has been shown that migration markers on T-cells are still intact following VZV infection which enables the virus to be transported to target sites (66). Interestingly, while there was still distinct EHV-1 ISH signal in the nucleus of the trigeminal ganglia at 70 dpi, no signal could be detected in non-neuronal cells at this time point. Moreover, there was a significant decrease in viral load when comparing TG 30 and 70 dpi. For HSV-1 infections, it has been shown that the number of T-cells in ganglia declines continuously between days 21 and 92 p.i. (16). Apoptosis of infected lymphocytes and degeneration of some neurons could be reason for the decrease of viral load in ganglia in the present study. However, it might also be evidence for the existence of a persistently infected EHV-1 memory T-cell pool, where T-cells are retrieved from ganglia, (re-)circulate in the blood and home to lymphoid tissue, where the virus remains stationary latent and/or starts new re-circulation. However, in our previous study no or only very low amounts of EHV-1 DNA was present in peripheral blood mononuclear cells (PBMC) at day 70 pi (unpublished data). This may imply that the majority of latently infected mononuclear cells are in tissue sites and it is challenging to detect very low amounts of silent virus in the circulating PBMCs using our methods as the collection time point represents a moment in time in the circulating latent infected cell pool. Further studies involving methods with higher sensitivity, e.g., Next Generation Sequencing, are needed to finally define the role and type of mononuclear cells in EHV-1 latency establishment.

Furthermore, latency in lymphatic tissue has been described as an advantage for the virus, as infected T-cells can enter rapidly the blood circulation to be transported to primary infection sites for replication, once reactivation occurs (19). In the present study, we report EHV-1 DNA in RLn and MesLn at 30 and 70 dpi. Respiratory associated lymphoid tissue has been previously described as latency location for EHV-1 (19–21, 67) and EHV-1 DNA could also be detected in MesLn and in the spleen (19, 23, 67). When comparing results 30 and 70 dpi, it was somewhat surprising, that the number of positive retropharyngeal lymph node samples decreased by 80% over time. While apoptosis of some EHV-1 infected T-cells is likely contributing to this decrease, positive T-cells could also migrate from lymph nodes toward neurons and/or other lymphatic tissue and therefore deplete positive signal in lymph nodes over time. A neurotropic re-circulation could also explain the findings in sympathetic trunk ganglia 70 dpi, where positive EHV-1 non-neuronal cells could still be detected.

In addition, while EHV-1 strain Ab4 is known to strongly dysregulate the host immune response by increasing inflammation and resulting in a high quantity of circulating infected cells, other EHV-1 strains may act differently in their immune evasive strategies (7, 68). Therefore, latency establishment and reactivation also may be strain dependent and more research is necessary to reveal the latently infected host pool in the populations worldwide.

Taken together, the present study confirms previous findings, identifying EHV-1 as both neurotropic and lymphotropic. EHV-1 most likely uses retrograde axonal transport as a direct pathway from the upper respiratory epithelium toward the TG, where latency is established in the nucleus of the neuron after a short replication cycle. Furthermore, we hypothesize, that T-cell tropism assists EHV-1 in its neurotropism and additionally provides latency establishment in lymphatic tissues other than respiratory associated lymphoid tissue. The navigation to neuronal structures throughout the body may enable the virus to establish latency in numerous ganglia, but further studies in random horse populations are required to elucidate if other neuronal structures than the TG can be repeatedly confirmed as alternative latency locations.

## Data Availability Statement

All datasets generated for this study are included in the article/supplementary material.

## Ethics Statement

The animal study was reviewed and approved by Michigan State University Institutional Animal Care and Use Committee.

## Author Contributions

KG and SS carried out the experiments and data analysis. KG wrote the manuscript with support from GSH and LG. GSH developed the experimental design of the horse infection experiment and conducted the clinical study. MK and DS conducted the necropsy and sample collection and GSH, KG, and LG contributed to sample collection. KM contributed to interpretation of histopathological and *in situ* hybridization results and performed figure formatting. FL and JS helped with the design and performance of the *in situ* hybridization experiment. FL, JS, and RF contributed to the interpretation of the results. LG supervised the project. All authors contributed to the article and approved the submitted version.

## Conflict of Interest

The authors declare that the research was conducted in the absence of any commercial or financial relationships that could be construed as a potential conflict of interest.
